# Colistin and Oxyclozanide co-loaded PLGA nano-microspheres to reverse colistin resistance can effectively treat colistin-resistant *Escherichia coli* infections

**DOI:** 10.1016/j.ijpx.2025.100402

**Published:** 2025-09-25

**Authors:** Shuai-hua Li, Meng-jing Feng, Hao-tian Shao, Jian-hua Liu, Hua Wu, Li Yuan, Xiao-yuan Ma, Gong-zheng Hu

**Affiliations:** Department of Pharmacology and Toxicology, College of Veterinary Medicine, Henan Agricultural University, Zhengzhou, China

**Keywords:** Colistin, Oxyclozanide, Resistance reversal, Co-loaded microsphere, *Escherichia coli* infection

## Abstract

Colistin (COL) is widely recognized as the last line of defense for treating MDR-negative bacterial infections, but currently, bacteria have a very serious resistance to COL. The combination of antibacterial drugs and adjuvant drugs can reverse drug resistance, enhance antibacterial activity, and improve therapeutic effects. It is currently regarded as a new safe and effective strategy for controlling drug resistance. In this study, we found that the combination of Oxyclozanide (OXY) and colistin can effectively reverse colistin resistance. For multiple colistin resistant *Escherichia coli* (*E. coli*) strains, COL-OXY-PLGA @MS significantly reduced the MIC of COL monotherapy (8 < MIC<64) by 40–160 times. The prepared COL-OXY-PLGA@MS had particle sizes of 140–160 nm, PDI of 0.03–0.2, COL loading of 5.14 % and OXY loading of 2.93 %. The release rate of COL in COL-OXY-PLGA@MS at 72 h was 39.31 %, and there was no burst release. Cytotoxicity assay, hemolysis test and long-term injection tests in mice have proved that COL-OXY-PLGA@MS has good safety and biocompatibility. It was clearly observed by SEM that the COL-OXY-PLGA@MS group disrupted *E. coli 58* cells under 1 h of action with obvious exudation of contents, and large number of cells ruptured at 4 h and 12 h. COL-OXY-PLGA@MS significantly reduced mortality rate after *E. coli* infection in mice. This study successfully prepared COL-OXY-PLGA@MS with high safety and strong antibacterial effect, which has great potential in the treatment of infections caused by color-resistant Gram-negative bacteria and provides a new and important strategy for the clinical application of colistin.

## Highlights

• This study demonstrated that a very low concentration of oxyclozanide could reverse bacterial colistin resistance, enhance the antibacterial activity of colistin.
• This study demonstrated the good biocompatibility of COL-OXY-PLGA@MS by both in vivo and in vitro methods.
• COL-OXY-PLGA@MS significantly reduces the MIC of colistin, significantly lowers the mortality rate of *Escherichia coli* infection in mice, and has achieved a new combination of colistin and adjuvant.

## Graphical abstract

Graphical Abstract

Schematic diagram of colistin and oxyclozanide co-loaded PLGA nano-microspheres and treatment regimen of colissin-resistant *E. coli* peritoneal infection in mice using COL-OXY-PLGA@MS. Create using biorender.com.

Insert Table of Contents artwork here

1. Characterization of the antibacterial activity of the combined use of colistin (COL) and oxyclozanide (OXY)

5. Encapsulation efficiency and Drug loading capacity

6. In Vitro and in Vivo Biocompatibility of COL-OXY-PLGA@MS

7. In Vitro bacteriostatic action of COL-OXY-PLGA@MS

8. In vivo antibacterial activity against colistin-resistant *E. coli* infection

**Figure:**
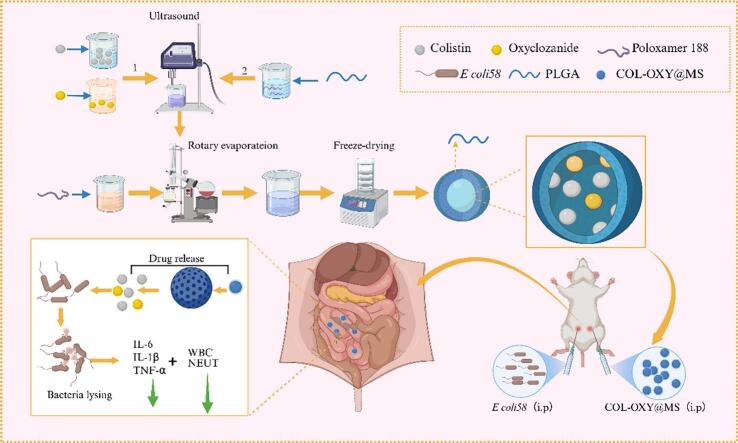

## 1 Introduction

Colistin (COL) is a cationic polypeptide antibiotic that has a powerful antibacterial effect on Gram-negative bacteria. It is the last line of defense for treating multi-drug resistant (MDR) Gram-negative bacterial infections that cannot be treated by other clinically available antibiotics (Zhao et al., 2023). In the treatment of infections caused by multidrug-resistant Gram-negative bacteria, colistin is irreplaceable by other clinically available antibiotics. However, the excessive use of colistin worldwide has led to a continuous increase in bacterial resistance rates, a phenomenon that has drawn significant attention from today's society (Global burden of bacterial antimicrobial resistance in 2019: a systematic analysis, 2022, LaPlante et al., 2018). The development of new antibacterial drugs with new targets to resist drug resistance is extremely difficult, involving high investment, long cycle and great risks. However, the combination of drugs, especially the combination of antibacterial drugs and adjuvants, can reverse drug resistance, enhance antibacterial activity and improve therapeutic effects. It is currently recognized as a safe, economical and effective strategy for controlling drug resistance.

Oxyclozanide (OXY) is a salicylamide anthelmintic drug. As a chemically synthesized drug against worm infections, it has excellent properties such as broad-spectrum, low toxicity, and low residue (Ayerbe-Algaba et al., 2019). In addition, this drug also has a good repellent and killing effect on tapeworms, which is also used in veterinary medicine for treating fluke infections in ruminants, exhibits activity against *Staphylococcus aureus*, *Clostridium difficile* and *Helicobacter pylori*, probably due to disruption of their cell envelope (Xu et al., 2019; Gedefaw, Mebratu, Dagnachew, and Fenta, 2025). And so far, there have been no reports of related drug resistance.

Previous studies have shown that the combination of colistin and Closantel (CST) has synergistic antibacterial activity against multidrug-resistant *Acinetobacter baumannii* (Tran et al., 2016). However, there are no studies on the dose–response relationship between CST and its reversal effect on colistin resistance. We attempted and found that salicylamide drugs such as Niclosamide and Rafoxanide can reverse the resistance of *Escherichia coli* (*E. coli*) and *Salmonella* to COL. and there is a special dose–response relationship between CST and its reversal effect on colistin resistance, which is not concentration-dependent. High reversal efficiency can be achieved within a low concentration range (Zhang et al., 2024; Cui et al., 2025). Meanwhile, we also discovered the same pattern when oxyclozanide was used in combination with COL, and this pattern is very applicable to the nano-drug delivery system.

Polylactide-*co*-glycolic acid (PLGA) is randomly polymerized from two monomers - lactic acid and glycolic acid. It is a degradable functional high-molecular organic compound with high biocompatibility, non-toxicity, and degradability. It can be used for targeted delivery of hydrophilic or hydrophobic drugs and is widely applied in the pharmaceutical, medical engineering materials, and modern industrial fields (Hua et al., 2021, Su et al., 2021).

This study aims to construct a drug delivery system of antagonizing drug-resistance, targeted, sustained-release, highly efficient and safe compound prescriptions based on the physicochemical properties and kinetic characteristics of COL and adjuvant drugs. PLGA microspheres, as a drug delivery system, overcome some of the shortcomings of traditional dosage forms and possess numerous advantages: (1) Multi-route administration: such as subcutaneous injection, intratumoral injection, intra-articular injection, and even oral administration (Wan, Bao, and Burgess, 2023; Chen et al., 2023); (2) Protective effect: they can encapsulate various molecules, providing antioxidant protection and enhancing their stability in vivo or in vitro (Jamaledin et al., 2020); (3) Long-acting sustained release: by effectively controlling drug release over a long period, it reduces the frequency of administration and improves medication safety (Blasi, 2019; Sharifi, Otte, Yoon, and Park, 2020); (4) Targeting effect: it can improve the tissue distribution of drugs, reduce drug toxicity, and enhance in vivo antibacterial activity (Yang, Mao, and Tan, 2020); (5) Good biocompatibility and degradability: PLGA has excellent biocompatibility, and its final hydrolysis products are H2O and CO2 (Lagreca et al., 2020); (6) Microsphere preparations can also mask the unpleasant odor of the encapsulated drugs and reduce their irritancy.

## 2 Materials and methods

### 2.1 Materials

All *E. coli* strains used in this study were collected and preserved in our laboratory. Porcine kidney cells (PK-15) were cultured in Dulbecco's modified Eagle medium. Colistin was purchased from Hebei Shengxue Dacheng Tangshan Pharmaceutical Co., LTD. (Hebei, China). Oxyclozanide was purchased from Shanghai MacLean Biochemical Technology Co., LTD. (Shanghai, China). PLGA was purchased from Jinan Jufukai Biotechnology Co., LTD. (Shandong, China). All the mice were from Liaoning Changsheng Biotechnology Co. LTD. (Liaoning, China).

### 2.2 Characterization of the antibacterial activity of the combined use of COL and OXY

The MIC of OXY and COL for the tested strains were determined using standard broth microdilution method according to CLSI guideline (CLSI, 2020). Using the above method, add colistin (2 mg/L) to the bacterial suspension to determine the OXY MIC of the tested strain, and define this OXY MIC as the minimum colistin resistance reversal concentration (MRC). Furthermore, following the previously reported protocol, the synergistic activity of OXY and COL against *E. coli* strains was evaluated by the checkerboard microdilution method. The fractional inhibitory concentration index (FICI) > 2 was defined as antagonism, 0.5 < FICI ≤2 was considered indifferent, and FICI ≤0.5 was defined as synergy (Cui et al., 2025).

### 2.3 Establishment of analytical methods before synthesis of COL-OXY-PLGA@MS

Determination of ultraviolet detection wavelength: prepare gradient concentration solutions of COL and OXY, and then use an ultraviolet spectrophotometer to detect the maximum absorption wavelengths of COL and OXY respectively within the wavelength range of 0-1000 nm.

Determination of standard curve: prepare gradient concentration solutions of COL and OXY, and then measure the absorbance of different concentrations of COL and OXY at 208 nm and 303 nm respectively. Then, plot a linear graph with the solution concentration as the abscissa and the absorbance as the ordinate to generate a standard curve.

The recovery rate of the analysis method was as follows: accurately weigh an appropriate amount of COL and OXY, dissolve them respectively with a certain amount of methanol, and then prepare three standard solutions of COL and OXY with four concentrations from high to low, namely 200 μg/mL, 100 μg/mL, 50 μg/mL, and 10 μg/mL respectively, using methanol. The absorbance values were determined respectively by ultraviolet-spectrophotometer. Then, the concentrations of COL and OXY were calculated respectively according to the regression equation of the standard curve, and the recovery rates and standard deviations were calculated.

The precision of the analytical method: Accurately weigh an appropriate amount of COL and OXY, dissolve them respectively with a certain amount of methanol, and then prepare three standard solutions of COL and OXY with low (10 μg/mL), medium (100 μg/mL), and high (200 μg/mL) concentrations using methanol. Then, conduct intra-day and inter-day precision tests respectively. The daily precision is measured by taking samples three times a day in the morning, at noon and in the evening to determine the absorbance value. The daily precision is measured for three days.

### 2.4 Synthesis and optimization of COL-OXY-PLGA@MS

**Scheme 1:**
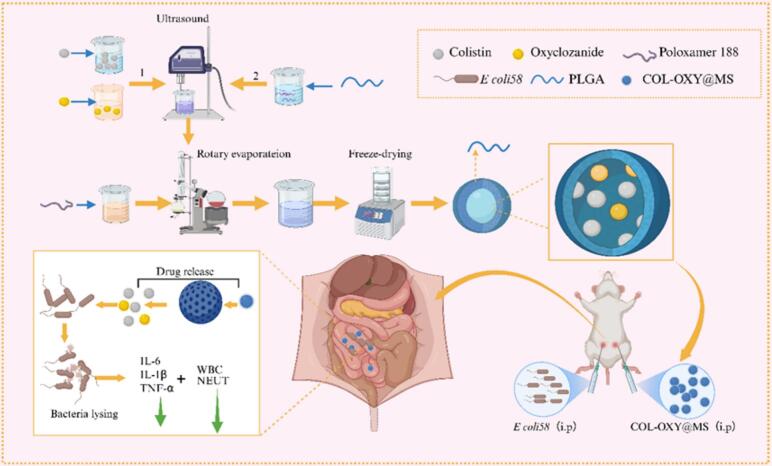
Schematic diagram of colistin and oxyclozanide co-loaded PLGA nano sustained release microspheres and treatment regimen of colistin-resistant *E. coli* peritoneal infection in mice using COL-OXY-PLGA@MS. Create using biorender.com.

Based on the preparation of microspheres by the W/O emulsification - solvent evaporation method [20], the process was improved and the W/O/W double emulsification-solvent evaporation method was used. Weigh the compound antibacterial drugs (COL 0.2 g and OXY 0.1 g), dissolve them respectively in water and ethanol, and mix the two to use as aqueous phase W1; Take an appropriate amount of PLGA and dissolve it in dichloromethane, with the final concentration set at 10 %, and use it as the oil phase O; Mix W1 and O, and perform ultrasound (130 W, 3 min) to form colostrum PE. Take 0.35 g of Poloxam P188 and dissolve it in 50 mL of distilled water as the aqueous phase W2; Under ultrasonic conditions (150 W, 3 min), PE was slowly dropped into W2 using a pipette, and microsphere emulsion was formed after ultrasonic treatment. Then dichloromethane is removed by vacuum evaporation at 40 °C. Finally, centrifuge at 3500 r/min for 10 min, collect the supernatant, and the microspheres are obtained (Scheme 1).

### 2.5 Characterization of COL-OXY-PLGA@MS

The COL-OXY-PLGA@MS was assessed for appearance, lyophilized mass, and resolvability. Additionally, transmission electron microscopy (TEM, Hitachi HT-7800, Japan) and scanning electron microscope (SEM, ZEISS Gemini SEM 300, German) were used to investigate the appearance for COL-OXY-PLGA@MS. The mean size, Zeta, and PDI of the COL-OXY-PLGA@MS were measured using the Zeta sizer Nano-ZSE (Zeta Sizer Nano series Nano-ZSE, Malvern, UK). A Nicolet iS20 infrared spectrometer (FTIR, Thermo Fisher Scientific, USA) was used to scan in the wave number range of 400–4000 cm^−1^to obtain the infrared spectra of each sample.

### 2.6 Encapsulation efficiency and Drug loading capacity

To determine the encapsulation efficiency (EE) and loading capacity (LC), the COL-OXY-PLGA@MS were lyophilized to measure the colistin content in the lyophilized NPs using UV spectrophotometry (UVS; Thermo, GENESYS 50) to calculate the EE and LC according to the following equations:

$$\mathrm{EE}\;\left(\%\right)=\text{Weight of colistin in}\;\mathrm{COL}-\mathrm{OXY}-\text{PLGA}@\mathrm{MS}/\text{total weight of added colistin}\times 100\%$$

$$\mathrm{LC}\;\left(\%\right)=\text{Weight of colistin in}\;\mathrm{COL}-\mathrm{OXY}-\text{PLGA}@\mathrm{MS}/\text{Weight of}\;\mathrm{COL}-\mathrm{OXY}-\text{PLGA}@\mathrm{MS}\times 100\%$$

### 2.7 In Vitro and in Vivo biocompatibility of COL-OXY-PLGA@MS

#### 2.7.1 Cytotoxicity assay

In vitro cytotoxicity was firstly determined by CCK-8 method. Pk-15 cells were incubated on 96-well plates in DMEM for 24 h. Subsequently, 100 μL of COL-OXY-PLGA@MS solution at different concentrations of 0.001, 0.01, 0.1, 1, and 10 mg/mL was added to a 96-well plate and incubated for 24 h at 37 °C. Next, 10 μL of CCK-8 solution was added to each well for coculturing for 4 h at 37 °C. The absorbance was measured at 540 nm and untreated cells were used as a negative control for 100 % cell viability. The cell viability was analyzed by calculating the percentage of the absorbance value of different concentrations of COL-OXY-PLGA@MS groups relative to the negative control group.

#### 2.7.2 Hemolysis test

The haemolytic activity of COL-OXY-PLGA@MS was determined according to the previous report (Anwer et al., 2019). Briefly, mice blood cells were treated with different concentrations of COL/OXY /COL-OXY-PLGA@MS(0–512 μg/mL) at 37 °C for 1 h as the experimental group. Normal saline treatment was used as the negative control group, and ultra-pure water treatment was used as the positive control group. Measure the absorbance of the supernatant at 540 nm and calculate the haemolysis of each sample by comparing it with a positive control.

#### 2.7.3 In vivo safety of COL-OXY-PLGA@MS in mice

The in vivo toxicity was determined using twelve healthy female Kunming mice (4-6 weeks old, weighing approximately 22–25 g). All animal experiments were performed in compliance with ARRIVE guidelines and were approved by the Ethics Committee for Experimentation at Henan Agricultural University. The mice were randomly divided into four groups, with 3 mice in each group. COL (5 mg/kg), OXY (5 mg/kg), PBS (0.1 mL), and COL-OXY-PLGA@MS (80 mg/kg, contains COL 4.112 mg/kg + OXY 2.344 mg/kg) were intraperitoneally injected respectively for 7 consecutive days, and the conditions of the mice were observed. Seven days later, one mouse was randomly selected from each group to take internal organ tissues and tissues for paraffin sections and HE staining to observe whether there were any pathological changes (Song et al., 2020). PBS was used as a positive control, and free COL and free OXY were used as controls to evaluate COL-OXY-PLGA@MS.

### 2.8 In Vitro bacteriostatic action of COL-OXY-PLGA@MS

#### 2.8.1 Minimal inhibitory concentration (MIC) assay

The MICs of the COL-OXY-PLGA@MS to the 5 colistin-resistant *E. coli* were measured by broth microdilution method according to the Clinical and Laboratory Standards Institute guidelines. All drugs were diluted using 2-fold serial dilutions in a sterile 96-well microtiter plate with Mueller-Hinton broth (MHB), and then, 100 μL of the bacterial suspensions (1 × 10^6^ CFU/mL) was added to each well. MIC values were defined as the lowest concentrations of drugs with no visible growth of bacteria after 16 h of incubation at 37 °C.

#### 2.8.2 Time-kill curves

To further investigate the bactericidal activity of the COL-OXY-PLGA@MS, the time-kill curves of randomly selected clinical col-resistant *E. coli* 58 were determined during the exponential growth phase. Bacterial cells at a final concentration of 1 × 10^5^ CFU/mL were cultured with MHB in OXY (128 μg/mL), COL (16 μg/mL), and the COL-OXY-PLGA@MS (containing COL and OXY at 0.36 and 0.184 μg/mL, respectively). Samples of the bacterial cells were collected after culturing for 0, 2, 4, 8, 12, and 24 h and spread on LB agar plates after dilution. CFUs were calculated after incubation at 37 °C overnight. All experiments were repeated three times on different days.

#### 2.8.3 Observation of bacterial morphology

To further investigate the effect of COL-OXY-PLGA@MS on bacteria, Antibacterial activity of the COL-OXY-PLGA@MS in vitro of randomly selected clinical col-resistant *E. coli* 58 were determined. Bacterial cells at a final concentration of 1 × 10^5^ CFU/mL were cultured with MHB in Conrtol(PBS), COL (the amount of COL contained in 1MIC COL-OXY-PLGA@MS), OXY (the amount of OXY contained in 1MIC COL-OXY-PLGA@MS), COL+OXY (The combined use of COL and OXY in 1MIC COL-OXY-PLGA@MS), Blank-COL-OXY-PLGA@MS, COL/OXY@MS (1MIC COL-OXY-PLGA@MS). Samples of the bacterial cells were collected after culturing for 1, 4, 12 h. CFUs were calculated after incubation at 37 °C overnight. All experiments were repeated three times on different days. The bacterial liquid was centrifuged at 2000 r/min for 5 min, then resuspended three times with PBS, fixed with 5 mL of 2.5 % glutaraldehyde, placed overnight at 4 °C, and the bacterial morphology of each group was observed using a scanning electron microscope. PBS was used as a positive control, and Blank-COL-OXY-PLGA@MS was used as a negative control.

### 2.9 In vivo antibacterial activity against colistin-resistant *E. coli* infection

Forty-eight female Kunming mice (6-8 weeks old, weighing approximately 22-25 g) were from Liaoning Changsheng Biotechnology Co. LTD. (Liaoning, China). All animal experiments were performed in compliance with ARRIVE guidelines and were approved by the Ethics Committee for Experimentation at Henan Agricultural University.

Prior to the experiments, the mice underwent a five days acclimatization period to their surroundings and all mice were provided ad libitum access to food. During the first three days of the adaptation period, all mice were given streptomycin through the stomach to reduce the interference of miscellaneous bacteria in the body. Subsequently, they were randomly divided into 8 groups: PBS treated group (healthy mice, no *E. coli 58*), positive control group (*E. coli 58* + PBS treated), free COL treated group, free OXY treated group, free COL+ free OXY treated group, COL-OXY-PLGA@MS-L treated group, COL-OXY-PLGA@MS-M treated group, COL-OXY-PLGA@MS-H treated group.

After the mice were fasted for 6 h, *E. coli 58* (2 × 10^8^ CFU/mL, 100 μL) was intraperitoneally injected into seven groups of mice to establish the intestinal membrane infection model in mice. Each group was intraperitoneally injected once a day for 2 days with the following methods: PBS(healthy mice, no *E. coli 58*), positive control (*E. coli 58* + PBS), free COL (5 mg/kg), free OXY (5 mg/kg), free COL+ OXY (COL 5 mg/kg, OXY 5 mg/kg), COL-OXY-PLGA@MS-L (10 mg/kg, contains COL 0.514 mg/kg + OXY 0.293 mg/kg), COL-OXY-PLGA@MS-M(50 mg/kg, contains COL 2.57 mg/kg + OXY 1.465 mg/kg), COL-OXY-PLGA@MS-H(80 mg/kg, contains COL 4.112 mg/kg + OXY 2.344 mg/kg). Healthy mice were injected with PBS as the blank control (marked as PBS), and the positive control group was also treated with PBS. Record the number of deaths of mice in each group within 7 days and calculate the survival rate.

Similarly, the only difference was that each mouse was challenged with a sublethal dose of *E. coli 58*(1 × 10^8^ CFU/mL) in the following tests. After treatment for 72 h, mice were sacrificed to obtain blood and major organs. Each group takes 1 mL of blood sample and sends it to Wuhan Servioebio Technology Co., Ltd.(China, Hubei) for blood routine testing to analyze the health level of blood cells. The blood was centrifuged at 4 °C to obtain serum, test liver function (alanine aminotransferase, aspartate aminotransferase, albumin) and renal function (urea, uric acid, creatinine) and use ELISA kits to measure the contents of IL-6, IL-1β and TNF-α in the serum. After the heart, liver, spleen, lung, kidney, small intestine and other tissues and organs of each group were removed, they were stored in 10 % formalin for more than 24 h, and then paraffin tissue sections were made for histological analysis. All the animal experiments were performed in compliance with the guidelines set by the National Research Council's Guide for the Care and Use of Laboratory Animals and approved by the Institutional Animal Care and Use Committee of Henan Agricultural University.

### 2.10 Statistical analysis

Statistical tests were performed using GraphPad Prism (Version 8.0). One-way analysis of variance (ANOVA) was used to compare differences between control and experimental groups. A significant level was set at 0.001, and *p* < 0.001 was considered statistically significant.

## 3 Results and discussions

### 3.1 Antibacterial activity of OXY in combination with colistin against col-resistant *E. coli* in vitro

Infectious diseases caused by MDR Gram-negative bacteria have posed a significant public health challenge due to the scarcity of effective treatments (Choi et al., 2024). Nowadays, colistin, as the last resort against multidrug-resistant bacterial infections, its clinical application has always been limited by plasmid-mediated or chromosome-mediated colistin resistance (Sheng et al., 2022). Improving the therapeutic effect and developing colistin adjuvants from approved drugs or functional foods is currently recognized as a safe, economical and effective strategy to control drug resistance in response to this crisis (Imai et al., 2020; Martin 2nd et al., 2020; Zhang et al., 2024). Although the literature has reported that this drug combination of Salicylamide derivatives enhances the antibacterial activity of COL against MDR Gram-negative bacteria (Cui et al., 2025; Copp et al., 2020; Yi et al., 2025), there are currently no reports on the combination of OXY and COL to combat MD Gram-negative bacteria.

**Fig. 1:**
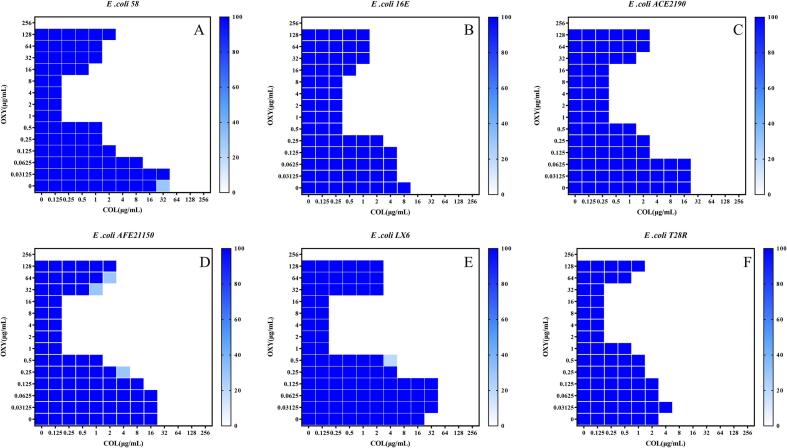
Representative heat plots of the microdilution checkerboard assay for the combination of colistin and OXY against colistin-resistant *E. coli*.

Checkerboard assays were used to evaluate the antibacterial activity of a combination of OXY and COL against col-resistant *E. coli*. Expected synergistic effect was observed in all test strains with FICI indices of≤0.5 (Table 1). Among them, the synergistic effect of colistin was the greatest when the concentration of OXY as 0.5–16 mg/L (Fig. 1). OXY robustly reversed COL resistance, with the MIC of COL decreased to a sensitivity of no more than 2 μg/mL, with FIC indices lower than 0.5 for all the tested resistant strains. Among all the tested strains, the MRC of OXY was between 0.25 and 4, indicating that a very low concentration of OXY could reverse bacterial COL resistance. These results demonstrated that the OXY can reverse the resistance phenotype of col-resistant *E. coli*. It is worth noting that in this study, we found that the combined use of OXY and COL demonstrated a synergistic effect, effectively enhancing the activity of colistin against Gram-negative bacteria with colistin resistance.

**Table 1:** Potentiation potency of oxyclozanide with colistin against colistin-resistant clinical *E. coli*.

Stain	COL- MIC	OXY- MIC	OXY- MRC	FICI	Interpretation
LX6	8	128	4	0.0625	synergy
AFE21150	64	128	4	0.0156	synergy
16E	16	64	2	0.0625	synergy
58	16	128	4	0.03125	synergy
HE2187	8	128	0.25	0.0468	synergy
PE77	16	64	2	0.0468	synergy
HFE88	8	128	2	0.125	synergy
ACE2190	32	64	4	0.0390	synergy
ACE21110	32	128	0.25	0.0390	synergy
116	32	128	1	0.0664	synergy

The table shows the MIC values of colistin and oxyclozanide against 10 colistin-resistant clinical *E. coli*. MRC, the minimum colistin resistance reversal concentration MRC. COL, colistin. OXY, oxyclozanide.

### 3.2 Establishment of analytical methods before synthesis of COL-OXY-PLGA@MS

#### 3.2.1 The maximum ultraviolet absorption wavelength and standard curve

The maximum ultraviolet absorption wavelengths of COL and OXY are 208 nm and 303 nm respectively (Fig. S1.A-B). The standard curves of COL and OXY are respectively described as follows (Fig. S1·C-D).

$$\mathrm{COL}\;\text{standard curve}:Y=0.0155\times +0.0123\;\left(R^{2}=0.9993\right)$$

$$\mathrm{OXY}\;\text{standard curve}:Y=0.0244\times -0.0433\;\left(R^{2}=0.9959\right)$$

#### 3.2.2 Test of the stability of the analytical method

The method for determining the loading capacity of COL-OXY-PLGA@MS was simple to operate, and the accuracy met the requirements. The average recovery rate of COL was 102.01 %, with RSD = 1.19 % < 2 %, and the average recovery rate of OXY was 101.98 %, with RSD = 0.50 < 2 %, indicating that the recovery rate of this method met the determination requirements (Table S1). The intraday precision (RSD) of the low, medium and high concentrations of the COL and OXY gradient solutions was all less than 2 %, and the RSD of the intraday precision was all less than 2 %, indicating good precision (Table S2, S3).

### 3.3 Synthesis and optimization of COL-OXY-PLGA@MS

COL-OXY-PLGA@MS was prepared by the W/O/W two-solvent-emulsification volatilization method. Polylactide-*co*-glycolic acid (PLGA) is a degradable functional high-molecular organic compound with good biocompatibility and the properties of capsule and film formation, and is non-toxic and it is widely applied in the fields of pharmaceuticals, medical engineering materials and modern industry (Bertilla, 2025). Then, the effects of drug ratio (COL: OXY, mass ratio), PLGA concentration, and poloxamer 188 concentration on COL-OXY-PLGA@MS particle size and PDI were screened by the single-factor method.

**Fig. 2:**
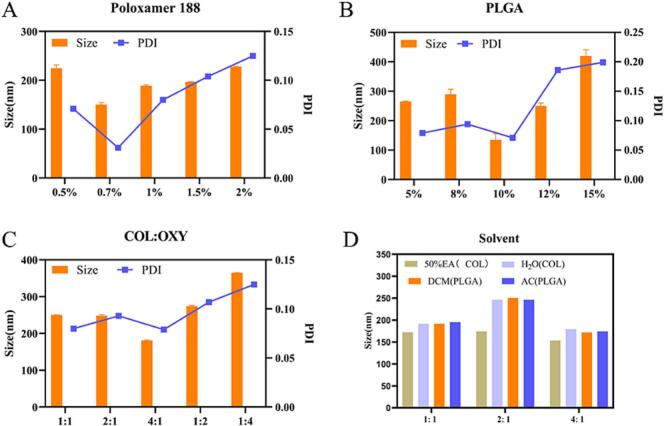
Synthesis and optimization of COL-OXY-PLGA@MS. (A) The effect of poloxamer 188 concentration on particle size and PDI of COL-OXY-PLGA@MS. (B) The effect of PLGA concentration on particle size and PDI of COL-OXY-PLGA@MS. (C) The effect of COL/OXY (mass ratio) on particle size and PDI of COL-OXY-PLGA@MS. (D) The effects of COL and PLGA solvents on COL-OXY-PLGA@MS particle size and PDI under different COL/OXY (mass ratio).

We found that when the drug ratio (COL: OXY) was 1:1, 2:1, 4:1, 1:2, and 1:4, the particle size was the smallest when the drug concentration was 4:1, followed by 2:1 (Fig. 2C). When the concentration of PLGA is 10 %, the particle size and PDI are the smallest (Fig. 2B). When the concentration of Poloxamer 188 was 0.7 %, the particle size of the microspheres and PDI were the smallest (Fig. 2A). Under the condition of the same drug ratio (COL: OXY), the particle size of COL dissolved in 50 % ethanol (50 % EA) is smaller than that in pure water. Since OXY has better solubility in anhydrous ethanol, we choose 50 % ethanol to dissolve COL. Under the condition of the same drug ratio (COL: OXY), the effects of dichloromethane and acetone as solvents for PLGA on particle size are very different (Fig. 2D).

The LC (%) amounts of COL and OXY in COL-OXY-PLGA@MS prepared by the optimized method were 5.14 % and 2.93 % respectively, and the EE (%) were 91.16 % and 97.52 % respectively. 1 g of COL-OXY-PLGA@MS freeze-dried powder contains 0.0514 g of col. and 0.0293 g of OXY.

### 3.4 Characterization of COL-OXY-PLGA@MS

**Fig. 3:**
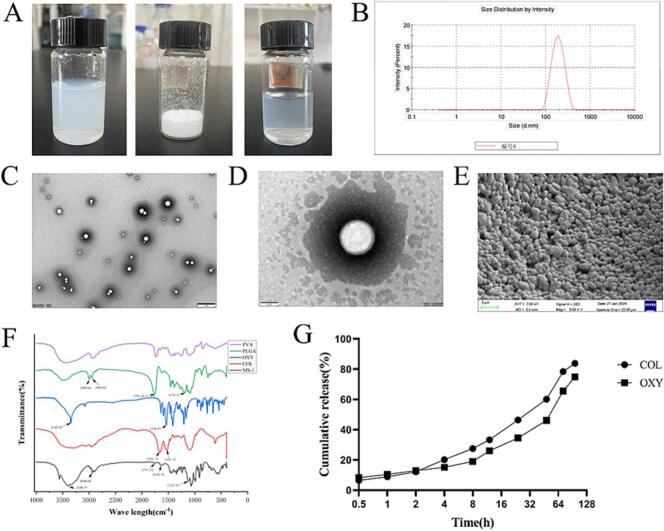
Characterization of COL-OXY-PLGA@MS. (A) From left to right are COL-OXY-PLGA@MS emulsion, COL-OXY-PLGA@MS freeze-dried powder, and COL-OXY-PLGA@MS freeze-dried powder reconstituted. (B) Particle size distribution. (C, D) (C, × 4000 and D, × 50,000) TEM images of COL-OXY-PLGA@MS. (E) (× 5000) SEM images of COL-OXY-PLGA@MS freeze-dried powder. (F) FTIR spectra of PVA, PLGA, OXY, COL, MS. (G) In vitro release of COL-OXY-PLGA@MS.

The COL-OXY-PLGA@MS were prepared according to the optimized formulation. The preparation process of COL-OXY-PLGA@MS is illustrated in Scheme 1. The states of its microsphere emulsion, freeze-dried powder and the reconstituted freeze-dried powder are shown in Fig. 3A. Morphology of the obtained COL-OXY-PLGA@MS was observed by scanning electron microscope (SEM) and transmission electron microscope (TEM). As shown in Fig. 3(C-E), the microspheres were spherical with smooth surfaces, complete shape and regular spherical form and had no aggregation. Moreover, the external membrane structure can be clearly observed, the sizes of the COL-OXY-PLGA@MS corresponded to the ones measured with Malvern laser particle size analyzer (PSA), as evident from Fig. 3B. The particle size of COL-OXY-PLGA@MS is 140–160 nm, the PDI is 0.05–0.2, and the sphericity is uniform, smooth and non-sticky, which is necessary for the microspheres with good release performance (Zhou et al., 2023). Thereafter, we observed that COL-OXY-PLGA@MS has a sustained-release function, this was probably due to the encapsulated drug inside the PLGA polymeric matrix being released by diffusion.

As shown in Fig. 3F, the COL spectrum (red) showed characteristic peaks at 1664.74 cm^−1^(−C=O amide bonds) and 1528.79 cm^−1^(−CH stretching). The characteristic FTIR spectrum of OXY (blue) signaling includes bending vibration of aromatic ring(C=C), −NO2 bending, and − N-H stretching bending, which occur at 1536.97 cm^−1^ and 3342.52 cm^−1^, respectively. The PLGA spectrum (rose red) showed characteristic peaks at 1179.71 cm^−1^(−C − O − C amide bonds) ， 1756.18 cm^−1^(−C=O stretching vibration),2998.64 and 2848.44 cm^−1^(−CH₃ and -CH₂ stretching vibration). Major peaks of the COL-OXY-PLGA@MS appeared at wave numbers 3389.37,2940.68,1753.67, 1629.01, and 1122.81 cm^−1^. These peaks of PLGA, COLand OXY were also observed in the prepared microspheres, confirming that no additional covalent bonds were formed during the preparation process.

In vitro release shows that the nano-microspheres have sustained-release function, Fig. 3G. The release rate of COL in COL-OXY-PLGA@MS was 6.512 % at 0.5 h, 18.35 % at 8 h, 21.73 % at 12 h, 28.42 % at 24 h, 32.29 % at 48 h, and 39.31 % at 72 h, respectively. The release of OXY in COL-OXY-PLGA@MS was 8.2825 % at 0.5 h, 11.65 % at 8 h, 17.53 % at 12 h, and 19.71 %, 23.01 % and 24.69 % at 24 h, 48 h and 72 h, respectively. The sustained release of drug might help in reducing the frequency of the drug's administration(Anwer et al., 2019).

### 3.5 In Vitro and in Vivo biocompatibility of COL-OXY-PLGA@MS

PLGA (Polylactic acid - Glycolic acid Copolymer) is a biodegradable polymer material that has been approved by the US Food and Drug Administration (FDA) for wide clinical use. Its degradation products are non-toxic and non-irritating to the human body and do not cause immune rejection or inflammatory reactions，and COL and OXY are commonly used drugs in clinical practice, it is necessary to evaluate the biocompatibility of COL-OXY-PLGA@MS both in vitro and in vivo.

#### 3.5.1 Cytotoxicity assay

**Fig. 4:**
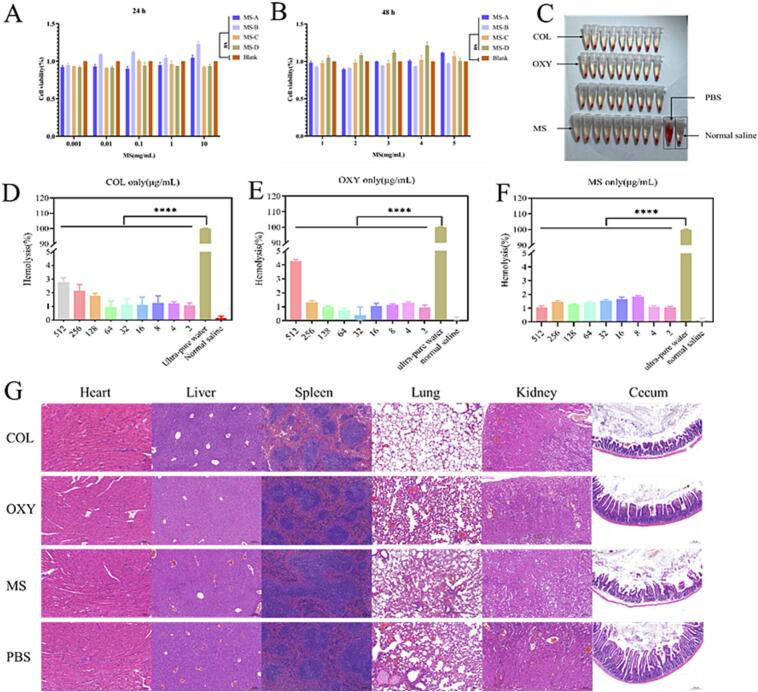
In Vitro and vivo Biocompatibility of COL-OXY-PLGA@MS. (A, B) Cytotoxicity assay of COL-OXY-PLGA@MS with 4 batches (A for 24 h and B for 48 h). The cell viability under different doses of COL-OXY-PLGA@MS was above 90 % at both 24 h and 48 h, indicating that the effect of COL-OXY-PLGA@MS on cell growth can be ignored. Data are expressed as mean ± SD, *n* = 3. (C) Hemolysis test of COL, OXY, MS, PBS and Nomal saline. (D—F) Hemolytic rate of COL, OXY and MS (2–512 μg/mL), respectively. The COL group, OXY group, COL-OXY-PLGA@MS group showed significant differences compared with the ultra-pure water group (****P < 0.0001). Data are expressed as mean ± SD, *n* = 3. (G) Heart, liver, spleen, lung, kidney and cecum, paraffin section (HE staining).

In addition, the cytocompatibility of COL-OXY-PLGA@MS toward the Porcine Kidney cell pk-15 was also evaluated through the CCK-8 assay, as nephrotoxicity is an issue with colistin treatment (Jafari and Elyasi, 2021). Cell viability of different groups at their effective dose exhibited no significant difference in 24 h and 48 h, suggesting that COL-OXY-PLGA@MS possessed negligible cytotoxicity (Fig. 4A-B).

#### 3.5.2 Hemolysis test

As COL-OXY-PLGA@MS is a drug mainly injection administration, the hemolysis of COL-OXY-PLGA@MS was first tested. The hemolysis results showed that both COL-OXY-PLGA@MS and free COL at their effective doses were not hemolytic, with a hemolysis level < 2 %, only OXY was slightly higher at 512 μg/mL, but still less than 5 %, indicating their hemocompatibility (Fig. 4 C—F).

#### 3.5.3 In vivo safety of COL-OXY-PLGA@MS in mice

Based on cytotoxicity tests and hemolysis tests, healthy Kunming mice were intraperitoneally injected with COL-OXY-PLGA@MS continuously for seven days. The biocompatibility of COL-OXY-PLGA@MS was further verified by changes in body weight and paraffin sections of internal organs. HE staining of paraffin sections showed that no obvious pathological changes occurred in the heart, liver, spleen, lung, kidney or small intestine of mice in the COL, OXY and COL-OXY-PLGA@MS groups at the given continuous dose (Fig. 4G). It is worth noting that this is already our maximum COL-OXY-PLGA@MS dose for the treatment of colistin-resistant *E. coli* in mice. It can also be seen from the changes in body weight of each group of mice that the growth trend of PBS in the administration group was almost the same. This also demonstrates that COL-OXY-PLGA@MS has good biocompatibility and that the prescribed dose is also a safe dose (Fig. S2, A-B).

The in vitro and in vivo biocompatibility of COL-OXY-PLGA@MS showed that COL-OXY-PLGA@MS had no significant effect on cell viability within 48 h, no effect on growth rate in mice after long-term injection, and no significant pathological changes in internal organs. This indicates that COL-OXY-PLGA@MS has good safety in the treatment of colistin-resistant *E. coli* infection in mice.

### 3.6 In Vitro bacteriostatic action of COL-OXY-PLGA@MS

#### 3.6.1 Minimal inhibitory concentration (MIC) assay

**Fig. 5:**
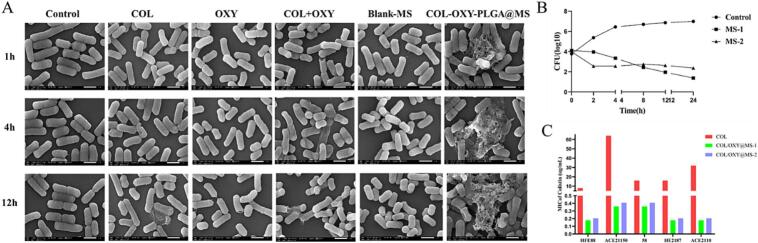
In Vitro bacteriostatic action of COL-OXY-PLGA@MS. (A) Scanning electron microscopic (SEM) images illustrating the morphological change of the bacterial cell membrane of *E. coli 58* after various treatments. (B) Time-kill curves of COL-OXY@MS; (C) MIC values of colistin, colistin in COL-OXY-PLGA@MS against different *E. coli* strains.

The MIC of COL and OXY as monotherapy was high for all five *E. coli* strains (Fig. 5C). COL-OXY-PLGA@MS-1 and COL-OXY-PLGA@MS-2 after PLGA encapsulation of COL and OXY significantly reduced the MIC of COL and OXY. However, we can find that the MIC of monotherapy measured by calculating the actual encapsulated doses of COL and OXY in COL-OXY-PLGA@MS-1 and COL-OXY-PLGA@MS-2 is not significantly reduced. Combined use of the actual dosage and proportion of COL and OXY in COL-OXY-PLGA@MS-1 and COL-OXY-PLGA@MS-2 simultaneously found that the MIC of COL and OXY against *E. coli* could be reduced to a certain extent. However, the effects are still significantly different from those of COL-OXY-PLGA@MS-1 and COL-OXY-PLGA@MS-2. The changes in these mic_s_ can prove that the COL-OXY-PLGA@MS-1 and COL-OXY-PLGA@MS-2 we prepared have good antibacterial effects. It can significantly reduce the MIC of COL and OXY against *E. coli*.

#### 3.6.2 Time-kill curves

To further evaluate the Antibacterial activity of the COL-OXY-PLGA@MS, we performed the time–kill curves assays using colistin-resistant *E. coli*. The results suggested that the COL-OXY-PLGA@MS exhibited excellent bactericidal efficacy. Specifically, neither alone COL nor OXY killed *E. coli 58* in the medium. However, the COL-OXY-PLGA@MS decreased CFUs of *E. coli 58* approximately by 1.5–3 log10 CFU/mL in 24 h compared to using any drug alone. Furthermore, the COL-OXY-PLGA@MS can enhance the bactericidal activity of COL compared with free COL+OXY (Fig. 5B).

#### 3.6.3 Antibacterial activity of the COL-OXY-PLGA@MS in vitro

The enhanced antibacterial activity of the COL-OXY-PLGA@MS was further confirmed by examining the bacterial surface morphology using scanning electron microscopy (SEM) (Fig. 5A). SEM images showed lytic bacterial cells destroyed and damaged by the COL-OXY-PLGA@MS treatment. In contrast, the cell membrane of bacterial cells remained relatively intact after treatment with the free drug combination or alone. Meanwhile, At the same time, the effects of drug sensitivity and in vitro against colistin-resistant *E. coli* demonstrated excellent antibacterial activity of COL-OXY-PLGA@MS, which was significantly much better than that of free COL and OXY and their combined use. As well as the Blank-MS without drug loading can eliminate the interference effect of carrier materials. In conclusion, the COL-OXY-PLGA@MS significantly enhance the antibacterial activity of the COL and OXY combination.

### 3.7 In vivo antibacterial activity against colistin-resistant *E. coli* infection

**Fig. 6:**
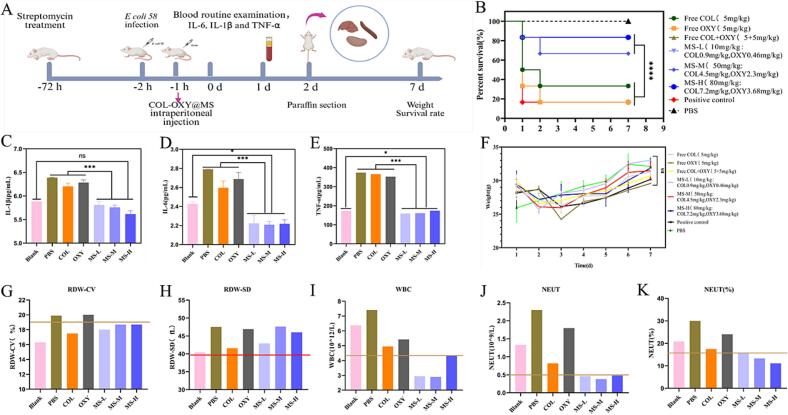
In vivo antibacterial activity against colistin-resistant *E. coli* infection. (A) Schematics of establishment of the *E. coli* infection mouse model and treatment schedule. Mice were infected with clinically isolated colistin-resistant *E. coli* isolate (2 × 10^8^ CFU/mL) via intraperitoneal injection followed by intraperitoneal injection of PBS, positive control, free COL (5 mg/kg), free OXY (5 mg/kg), free COL+ OXY (COL 5 mg/kg, OXY 5 mg/kg), COL-OXY-PLGA@MS-L (10 mg/kg, contains COL 0.514 mg/kg + OXY 0.293 mg/kg), COL-OXY-PLGA@MS-M(50 mg/kg, contains COL 2.57 mg/kg + OXY 1.465 mg/kg), COL-OXY-PLGA@MS-H(80 mg/kg, contains COL 4.112 mg/kg + OXY 2.344 mg/kg) at 1 h postinfection. Mice were sacrificed to extract the blood and major organs to determine the pathological change. Another batch of mice was retained until 7d to monitor the survival rate after different treatments. (B, F) Survival rate and weight of infected mice after treatment with PBS(*n* = 6), positive control, free COL (*n* = 2), free OXY (*n* = 1), free COL+ OXY (*n* = 4), COL-OXY-PLGA@MS-L (n = 2), COL-OXY-PLGA@MS-M(n = 4), COL-OXY-PLGA@MS-H(*n* = 5). COL-OXY-PLGA@MS-M and COL-OXY-PLGA@MS-H significantly improved the survival rate of mice (*p* < 0.0001), with survival increasing from 33.33 % (COL) to 66.67 % and 83.33 %, respectively. There was a significant difference among the COL-OXY-PLGA@MS (MS-L/M/H) group and the free COL group, the free OXY group, and the free COL+OXY group (****P < 0.0001). (C-E) IL-6, IL-1β and TNF-α after treatment with PBS, positive control, free COL (5 mg/kg), free OXY (5 mg/kg), free COL+ OXY (COL 5 mg/kg, OXY 5 mg/kg), COL-OXY-PLGA@MS-L (10 mg/kg), COL-OXY-PLGA@MS-M(50 mg/kg) in the blood. COL-OXY-PLGA@MS demonstrated the ability to reduce the inflammatory response. The levels of TNF-α, IL-1β, and IL-6 in the serum of mice treated with COL-OXY-PLGA@MS-L, COL-OXY-PLGA@MS-M, and COL-OXY-PLGA@MS-H did not increase. There was no significant difference compared with blank mice. Statistical analysis was conducted using the student's *t*-test. ns, no significance; *, *p* < 0.1, **, *p* < 0.01; ***, *p* < 0.001; ****, *p* < 0.0001. (G-K) Blood routine test results after treatment of the mouse colistin-resistant *E. coli* infection model.

To investigate the antibacterial activity and therapeutic potential of COL-OXY-PLGA@MS in vivo, a mouse model of abdominal infection was established through the injection of clinically isolated col-resistant *E. coli* (Fig. 6A).

#### 3.7.1 Survival rate

Mice were infected with clinically isolated colistin-resistant *E. coli 58* isolate (2 × 10^8^ CFU/mL) via intraperitoneal injection followed by intraperitoneal injection of PBS, free COL, free OXY, free COL+ OXY, COL-OXY-PLGA@MS-L, COL-OXY-PLGA@MS-M, COL-OXY-PLGA@MS-H at 1 h postinfection. As shown in Fig. 6B, treatment with alone OXY and COL did not exert adequate protection in the infected mice. Excitingly, administration of COL-OXY-PLGA@MS-M and COL-OXY-PLGA@MS-H significantly improved the survival rate of mice, with survival increasing from 33.33 % (COL) to 66.67 % and 83.33 % (33.34 %–50 %, *p* < 0.0001), respectively. Although COL-OXY-PLGA@MS-M (contains COL 2.57 mg/kg + OXY 1.465 mg/kg) had comparable protective effects on mice as free COL+OXY (COL 5 mg/kg + OXY 5 mg/kg), COL-OXY-PLGA@MS-M had lower drug content and could reduce the frequency of administration. And the weight of each group was showed in Fig. 6F.

#### 3.7.2 Anti-inflammatory action

After centrifuging the blood of mice in each group to obtain serum, the contents of TNF-α, IL-1β and IL-6 were determined by Mouse IL-6 ELISA KIT, TNF-α ELISA KIT, Mouse IL-1β ELISA KIT (Shanghai Yuanju Biotechnology Center). It was found that the contents of TNF-α, IL-1β and IL-6 in the serum of mice treated with intraperitoneal injection of PBS, free COL and free OXY in the mouse *E. coli 58* peritoneal infection model were significantly increased. It is notable that the levels of TNF-α, IL-1β and IL-6 in the serum of mice treated with COL-OXY-PLGA@MS-L, COL-OXY-PLGA@MS-M and COL-OXY-PLGA@MS-H were not increase, and there was no significant difference compared with Blank mice (Fig. 6C-E).

#### 3.7.3 Pathology test of tissues

**Fig. 7:**
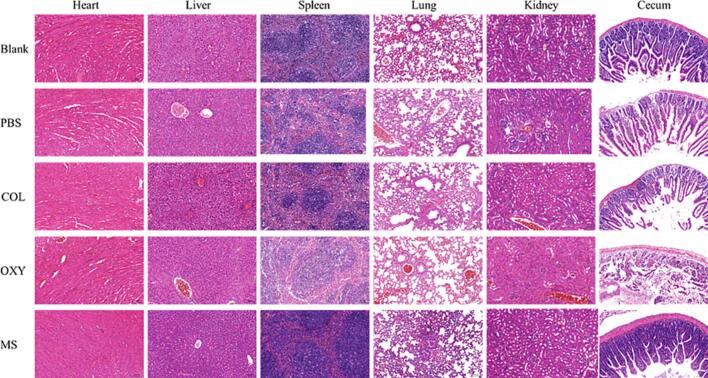
Visceral tissue sections of mouse models of colistin-resistant *E. coli* infection treated with PBS, free COL, free OXY and COL-OXY-PLGA@MS.

**Fig. 8:**
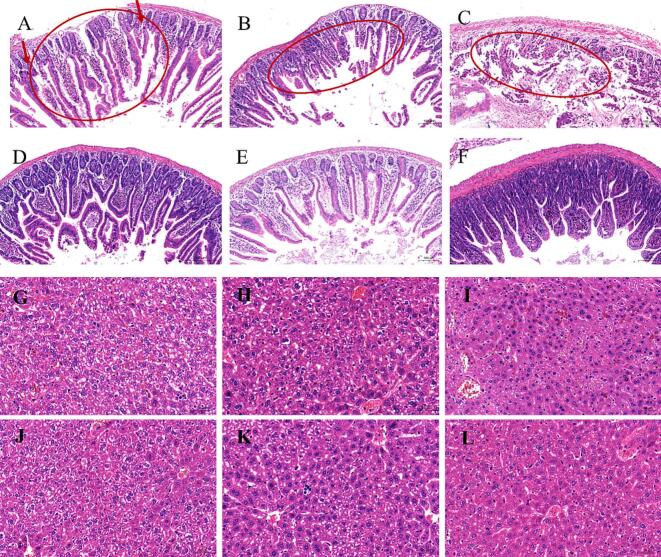
Paraffin sections of the small intestine and liver after treatment of the mouse colistin-resistant *E. coli* infection model, stained with HE (20×). A-F is the small intestine section diagram, and G-L is the liver section diagram. (A, G) positive control group. (B, H) COL group. (C, I) OXY group. (D, J) PBS group. (E, K) COL-OXY-PLGA@MS-L group. (F, L) COL-OXY-PLGA@MS-M group.

After treatment in each group of *E. coli* infected mice, by observing the tissues such as the heart, liver, spleen, lung, kidney and small intestine, it could be found that there were no obvious pathological changes in the HE sections of the heart, lung and kidney, as shown in Fig. 7. However, in the positive control group, the COL group, and the OXY group, obvious villi rupture and injury were observed in the small intestine, and there were bleeding points on the intestinal wall, while the intestine after COL-OXY-PLGA@MS treatment was no different from normal (Fig. 8A-F). Another notable point was that in the positive control group, COL group, and OXY group of mice, hepatocyte cords were disordered in some areas of the liver, the intrinsic structure of the hepatic lobules was lost, and hepatocytes were swollen. There was almost no difference between the COL-OXY-PLGA@MS treatment group and the blank group (Fig. 8G-L).

#### 3.7.4 Blood routine examination

RBC, HGB and MCHC fluctuated in the COL group, OXY group and COL-OXY-PLGA@MS group, all within the normal range (Fig. S3). WBC and NEUT were significantly increased in the PBS group, COL group and OXY group, but not in the COL-OXY@MS group (Fig. 6G-K).

This also indicated that our preparation of COL-OXY-PLGA@MS did enhance the antibacterial activity of COL. COL-OXY-PLGA@MS significantly improved the survival rate of colistin-resistant *E. coli* infection models in mice, significantly reduced the levels of IL-6, IL-1β, TNF-α in serum, and effectively protected against *E. coli* liver and intestinal injury. In other words, the COL-OXY-PLGA@MS exerted good protection and therapeutic effects on intestinal infections.

## 4 Conclusions

In this study, we found that OXY can effectively reverse colistin resistance, enhance colistin antibacterial activity, and successfully prepare COL-OXY-PLGA@MS through an optimized W/O/W double emulsion method, providing a new and important basis for the clinical application of COL. These COL-OXY-PLGA@MS exhibited an appropriate particle size distribution, demonstrating homogeneous and spherical characteristics. Additionally, they demonstrated a slow-release effect. The results of our in vitro and in vivo experiments confirmed that the COL-OXY-PLGA@MS exhibited potent antibacterial effects and it also has a relatively high level of security. Therefore, the application of COL-OXY-PLGA@MS has great potential in the treatment of infections caused by chromogenic gram-negative bacteria, and it also develops new clinical applications of colistin.

## Funding

This study was financed by the 10.13039/501100012166National Key Research and Development Program of China (2023YFD1800105) and the 10.13039/501100001809National Natural Science Foundation of China (32373069).

## CRediT authorship contribution statement

**Shuaihua Li:** Writing – original draft, Validation, Methodology, Data curation. **Meng-jing Feng:** Writing – original draft, Software, Investigation, Visualization. **Hao-tian Shao:** Writing – original draft, Software, Investigation. **Jian-hua Liu:** Conceptualization, Formal analysis. **Hua Wu:** Conceptualization, Formal analysis. **Li Yuan:** Conceptualization, Formal analysis. **Xiao-yuan Ma:** Writing – review & editing, Supervision, Project administration. **Gong-zheng Hu:** Conceptualization, Resources, Funding acquisition, Project administration.

## Ethics approval and consent to participate

All animal experiments have been approved by the Animal Ethics Committee of Henan Agricultural University. The study was conducted in accordance with the local legislation and institutional requirements.

## Consent for publication

Not applicable.

## Declaration of competing interest

The authors declare that they have no competing interests.

## Data Availability

Data will be made available on request.

## Appendix A Supplementary data

Supplementary material Image 1
