# Supplementary material for: Colistin and Oxyclozanide co-loaded PLGA nano-microspheres to reverse colistin resistance can effectively treat colistin-resistant Escherichia coli infections
Source: Int J Pharm X. 2025 Sep 25;10:100402. doi: 10.1016/j.ijpx.2025.100402 (PMC12516539; doi:10.1016/j.ijpx.2025.100402)
Supplement: Supplementary file 1 — Supplementary material [file mmc1.docx]

**Supplementary material**

**Tables**

Table S1 Recovery rates of analytical methods for COL and OXY

|  | Concentration（μg/ml） | | Average  absorbance value | Recovery (%) | Average  recovery (%) | RSD（%） |
| --- | --- | --- | --- | --- | --- | --- |
| COL | | 10 | 0.172 | 103.30 | 102.01 | 1.19 |
|  |  | 50 | 0.794 | 100.91 |  |  |
|  |  | 100 | 1.573 | 100.69 |  |  |
|  |  | 200 | 3.365 | 103.15 |  |  |
| OXY | | 10 | 0.217 | 101.8 | 101.98 | 0.50 |
|  |  | 50 | 1.208 | 102.50 |  |  |
|  |  | 100 | 2.456 | 102.40 |  |  |
|  |  | 200 | 4.901 | 101.23 |  |  |

The average recovery rate of COL was 102.01%, with RSD=1.19%<2%, and the average recovery rate of OXY was 101.98%, with RSD=0.50<2%, indicating that the recovery rate of the ultraviolet analysis method met the determination requirements.

Table S2 Intra-day and inter-day precision of COL

|  | Concentration（μg/ml） | Time | In-day precision | | | Inter-day precision | | |  |
| --- | --- | --- | --- | --- | --- | --- | --- | --- | --- |
|  |  |  | OD | Average | RSD (%) | | Average | RSD (%) | |
| COL | 10 | Day 1 | 0.157  0.170  0.146 | 0.157 | 0.98 | | 0.161 | 1.56 | |
|  |  | Day 2 | 0.190 | 0.180 | 0.78 | |  |  |  |
|  |  |  | 0.179 |  |  |  |  |  |  |
|  |  |  | 0.171 |  |  |  |  |  |  |
|  |  | Day 3 | 0.142 | 0.147 | 0.54 | |  |  |  |
|  |  |  | 0.155 |  |  |  |  |  |  |
|  |  |  | 0.146 |  |  |  |  |  |  |
|  | 100 | Day 1 | 1.620  1.648  1.614 | 1.627 | 1.48 | | 1.636 | 1.59 | |
|  |  | Day 2 | 1.614 | 1.629 | 1.17 | |  |  |  |
|  |  |  | 1.633 |  |  |  |  |  |  |
|  |  |  | 1.642 |  |  |  |  |  |  |
|  |  | Day 3 | 1.653 | 1.652 | 0.37 | |  |  |  |
|  |  |  | 1.648 |  |  |  |  |  |  |
|  |  |  | 1.657 |  |  |  |  |  |  |
|  |  | Day 1 | 2.477 | 2.454 | 1.58 | |  |  |  |
|  |  |  | 2.442 |  |  |  | 2.457 | 1.43 | |
|  | 200 |  | 2.445 |  |  |  |  |  |  |
|  |  | Day 2 | 2.455 | 2.450 | 1.00 | |  |  |  |
|  |  |  | 2.459 |  |  |  |  |  |  |
|  |  |  | 2.436 |  |  |  |  |  |  |
|  |  | Day 3 | 2.458 | 2.468 | 0.90 | |  |  |  |
|  |  |  | 2.467 |  |  |  |  |  |  |
|  |  |  | 2.480 |  |  |  |  |  |  |

The intraday precision (RSD) of the low, medium and high concentrations of the COL gradient solutions was all less than 2%, and the RSD of the intraday precision was all less than 2%, indicating good precision.

Table S3 Intra-day and inter-day precision of OXY

|  | Concentration(μg/ml) | Time | In-day precision | | | Inter-day precision | | |  |
| --- | --- | --- | --- | --- | --- | --- | --- | --- | --- |
|  |  |  | OD | Average | RSD (%) | | Average | RSD (%) | |
| OXY | 10 | Day 1 | 0.219  0.232  0.239 | 0.230 | 0.83 | | 0.240 | 1.45 | |
|  |  | Day 2 | 0.237 | 0.252 | 1.13 | |  |  |  |
|  |  |  | 0.256 |  |  |  |  |  |  |
|  |  |  | 0.264 |  |  |  |  |  |  |
|  |  | Day 3 | 0.233 | 0.239 | 0.4 | |  |  |  |
|  |  |  | 0.243 |  |  |  |  |  |  |
|  |  |  | 0.242 |  |  |  |  |  |  |
|  | 100 | Day 1 | 2.437  2.441  2.452 | 2.443 | 0.63 | | 2.445 | 0.70 | |
|  |  | Day 2 | 2.437 | 2.444 | 0.57 | |  |  |  |
|  |  |  | 2.445 |  |  |  |  |  |  |
|  |  |  | 2.451 |  |  |  |  |  |  |
|  |  | Day 3 | 2.441 | 2.447 | 0.83 | |  |  |  |
|  |  |  | 2.459 |  |  |  |  |  |  |
|  |  |  | 2.442 |  |  |  |  |  |  |
|  |  | Day 1 | 3.389 | 3.375 | 1.10 | |  |  |  |
|  |  |  | 3.362 |  |  |  | 3.364 | 1.45 | |
|  | 200 |  | 3.374 |  |  |  |  |  |  |
|  |  | Day 2 | 3.358 | 3.352 | 1.11 | |  |  |  |
|  |  |  | 3.336 |  |  |  |  |  |  |
|  |  |  | 3.361 |  |  |  |  |  |  |
|  |  | Day 3 | 3.376 | 3.365 | 1.05 | |  |  |  |
|  |  |  | 3.351 |  |  |  |  |  |  |
|  |  |  | 3.369 |  |  |  |  |  |  |

The intraday precision (RSD) of the low, medium and high concentrations of the OXY gradient solutions was all less than 2%, and the RSD of the intraday precision was all less than 2%, indicating good precision.

**Figures**


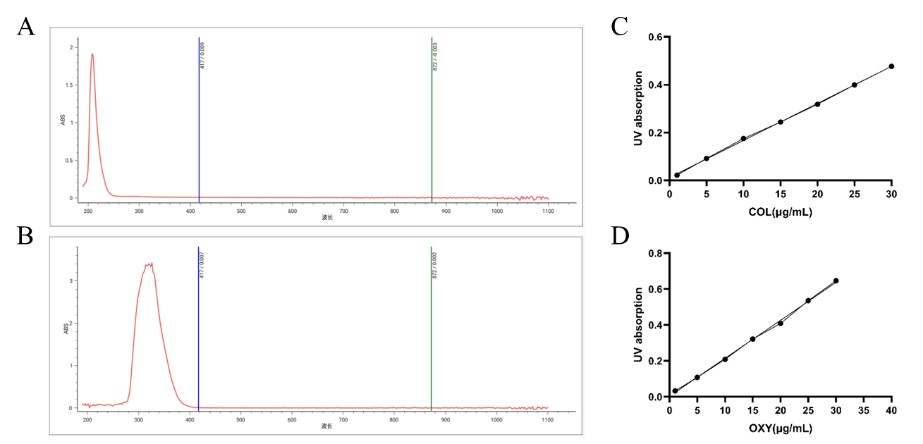


Figure S1. Establishment of the analytical method. (A, B) The maximum absorption wavelengths of COL and OXY, respectively. (C, D) The standard curve of COL and OXY, respectively.


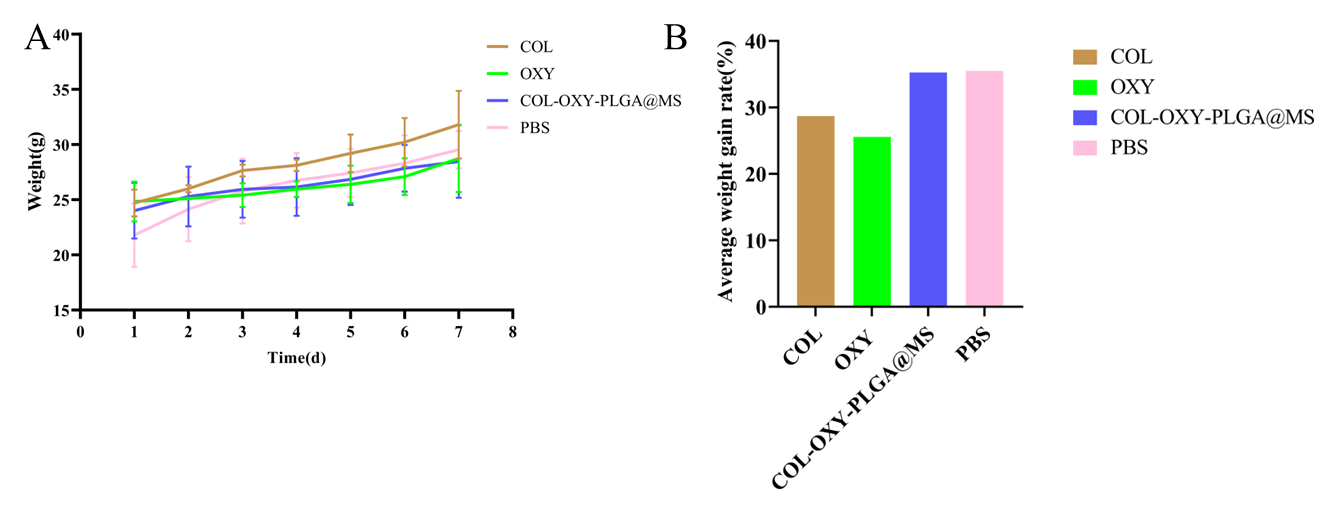


Figure S2. In vivo safety of COL-OXY-PLGA@MS in mice. (A) The trends in weight gain observed in the COL, OXY, COL-OXY-PLGA@MS and PBS groups. (B) The Average weight gain rate of COL, OXY, COL-OXY-PLGA@MS and PBS groups.


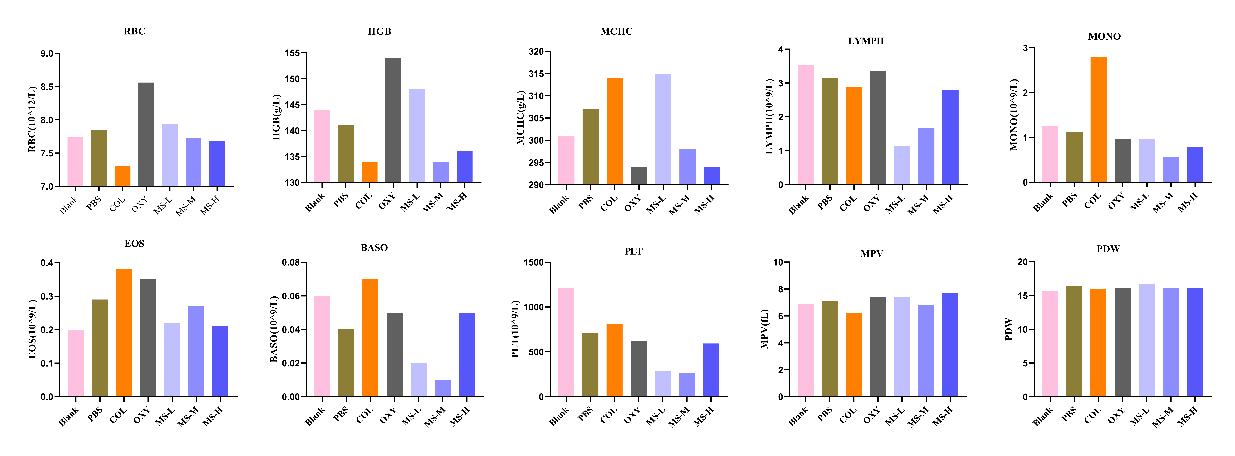


Figure S3. Blood routine test results after treatment of the mouse colisin-resistant *E. coli* infection model.
